# Development of a Novel Thyroid Function Fluctuated Animal Model for Thyroid-Associated Ophthalmopathy

**DOI:** 10.1371/journal.pone.0148595

**Published:** 2016-02-12

**Authors:** Yunhai Tu, Yilong Wang, Luna Ding, Jiao Zhang, Wencan Wu

**Affiliations:** 1 The Eye Hospital of Wenzhou Medical University, Wenzhou, 325000, PR China; 2 Department of Laboratory Animal Center, Wenzhou Medical University, Wenzhou, 325000, PR China; University of Florida, UNITED STATES

## Abstract

**Background:**

The establishment of a suitable and stable animal model is critical for research on thyroid-associated ophthalmopathy (TAO). In clinical practice, we found that patients treated with I-131 often exhibit TAO; therefore, we aimed to establish a novel thyroid function fluctuated animal model of TAO by simulating the clinical treatment process.

**Methods:**

We treated SD rats with I-131 to damage the thyroid and then used sodium levothyroxine (L-T4) to supplement the thyroid hormone (TH) levels every seven days, leading to a fluctuating level of thyroid hormones that simulated the status of clinical TAO patients. Rats administered normal saline were considered as a control. The weight, intraocular pressure, and serum T3, T4, TSH and TRAb levels of the rats were measured, and the pathological changes were analyzed by H&E staining and transmission electron microscopy (TEM).

**Results:**

The experimental rats (TAO group) exhibited significantly reduced weight and elevated intraocular pressure compared with the control rats. Meanwhile, the serum levels of T3 and T4 were up-regulated in the TAO group, but the TSH level decreased during the 10-week study. Moreover, increased numbers of blood vessels and inflammatory cell infiltrations were observed in the orbital tissues of the TAO rats, while no abnormal changes occurred in the control rats. The orbital myofibrils in the TAO rats appeared fractured and dissolved, with twisted structures. Mitochondrial swelling and vacuoles within the endoplasmic reticulum, swelling nerve fibers, shedding nerve myelin, and macrophages were found in the TAO group.

**Conclusion:**

Rats treated with I-131 and sodium levothyroxine exhibited characteristics similar to those of TAO patients in the clinic, providing an effective and simple method for the establishment of a stable animal model for research on the pathogenesis and treatment of TAO.

## Introduction

Thyroid-associated ophthalmopathy (TAO) is an autoimmune disorder of the orbit that is closely related to Graves’ disease [1], which has a high morbidity rate in adult patients with orbital disease. TAO is usually associated with hyperthyroidism symptoms, and some Hashimoto's thyroiditis patients with hypothyroidism or normal thyroid function can have TAO [1, 2]. Clinical TAO patients may develop eyelid retraction, proptosis, exposure keratitis and corneal ulcers with obvious pain, photophobia, tearing, diplopia and eyeball movement disorder symptoms, which is sometimes even accompanied by visual neuropathy [3, 4]. The orbital tissues, particularly the extraocular muscles and retro-orbital fat tissue, are two major sites of involvement in thyroid-associated ophthalmopathy (TAO). Edema and lymphocytic infiltration in these tissues are the prominent histological features of TAO [1–6]. However, until now, the initiative event and primary autoantigens for lymphocyte homing to the orbit and pathogenesis of TAO remain unclear [1]. Therefore, it is important to establish a stable animal model for research on the pathogenesis and prevention of TAO in the clinic.

The methods employed to establish an animal model of TAO include the use of pituitary extracts combined with thyroidectomy to treat the animal, thyrotropin receptor (TSHR) peptide or nucleic acid immunization, injection of TSHR-transfected cells or TSHR-activated T cells into experimental animals, and multi-gene co-immunization of animals [7–10]. These animal models can produce the TSHR antibody (TRAb) and exhibit some of the symptoms of thyroiditis and eye disease [7, 10]; however, differences between human TAO and animal models are still evident, limiting their application in related studies. Thus, it is valuable to establish a novel and stable animal model with similar changes in serum thyroxine levels and ocular change characteristics.

Early in 1992, Tallstedt et al. [11] reported that hyperthyroidism patients treated with I-131 are more prone to developing TAO in comparison to other therapies. Bartalena et al. and Traisk et al. [12, 13] stated that the occurrence and severity of Graves’ ophthalmopathy (GO) are closely related to radioactive iodine treatment and are accompanied by an increased level of TRAb. Eckstein et al. [14, 15] showed that the TBII level (TSH binding inhibitory immunoglobin) is elevated in severe GO cases. In clinical practice, we observed that hyperthyroidism patients treated with I-131 often exhibit hypothyroidism, TAO and a higher TRAb level, which is consistent with the above research. Therefore, in the present study, we aimed to establish a novel animal model of TAO by simulating the clinical TAO process of thyroid hormone fluctuation. Rats were first administered I-131 to induce hypothyroidism, followed by treatment with sodium levothyroxine (L-T4) to replenish the thyroid hormone levels at different time points. The eye changes of the model were assessed and evaluated.

## Experimental Procedures

### 2.1 Animal treatment and grouping

All animal procedures were approved by the Wenzhou Medical University Animal Care and Use Committee, which is certified by the Chinese Association of Accreditation of Laboratory Animal Care. Total Thirty healthy Sprague-Dawley (SD) rats (8–10 weeks and 250–300 g in weight) were provided by the Wenzhou Medical University Laboratory Animal Center. The rats were separated into groups of 5 in plastic cages with stainless steel mesh lids in a ventilated room, which was maintained at 20±2°C and 60%±10% humidity under a 12-h light-dark cycle. All rats had free access to food and water. The rats were given I-131 at a dose of 400 mCi by intragastric administration, leading to damage of the thyroid. The rats were then administered sodium levothyroxine (L-T4) by gavage at a dose of 200 μg/100 g to supplement the thyroid hormone (TH) levels every seven days (i.e., the 2nd, 4th, 6th, 8th, and 10th weeks). Thus, the TH levels fluctuated in these rats, which are referred to as the fluctuation group (F group) in this study. Another 15 rats, which were considered as the control, were given normal saline by gavage when the rats of the F group were administered I-131 or L-T4.

### 2.2 Monitoring of weight and intraocular pressure

During the experimental period (10 weeks), the weight of each rat was measured every week at the same time. The intraocular pressure of both eyes was also monitored each week using an ophthalmotonometer. Before the measurement, 10% chloral hydrate (300 mg/kg) was used to anesthetize the rats.

### 2.3 Measurement of serum levels of T3, T4, TSH and TRAb

Before blood was drawn, the rats were anesthetized by 10% chloral hydrate (300 mg/kg). Blood was drawn for hematology and serum biochemistry analysis using a standard vein blood collection technique each week during the experimental period. Total serum-free T3, T4 and TSH levels were measured in duplicate using a commercially available competitive radio immunity assay (RIA) according to the manufacturer’s instructions. The TRAb level was monitored by radio receptor assay (RRA).

### 2.4 Orbital tissue histological characterization

The rats were sacrificed by overdose injection of 10% chloral hydrate, and the orbital tissues were carefully dissected. The samples were immediately fixed in paraformaldehyde and then embedded in paraffin. Next, the tissues were cut into 5-μm sections for histopathological examination by hematoxylin-eosin (H&E) staining. Briefly, the paraffin-embedded sections were de-paraffinized and rehydrated and were then washed with water and subjected to H&E staining. All of the sections were then observed using a microscope.

### 2.5 TEM observation of orbital tissue

For subcellular observation, orbital tissues were excised and immediately fixed in 2.5% glutaraldehyde for 24 h; the samples were then treated according to the general protocols for TEM observation. Briefly, the samples were fixed with 1% osmium tetroxide for 2 h, routinely dehydrated through a graded ethanol series, and embedded in epoxy resin. The resin-embedded blocks were cut into 60- to 80-nm ultrathin sections using an ultra-microtome. The ultrathin sections were placed on carbon-coated nickel grids and examined with an H-600 TEM (H-600; Tokyo, Japan) operating at 80 kV.

### 2.6 Statistical analysis

The data were described as the mean ± standard deviation (mean ± SD). The differences between means were statistically analyzed by one-way ANOVA with a post-hoc test using Tukey’s method (GraphPad Prism 5). Differences with a *p* value of less than 0.05 were considered significant.

## Results

### 3.1 Gross observation

**Fig 1:**
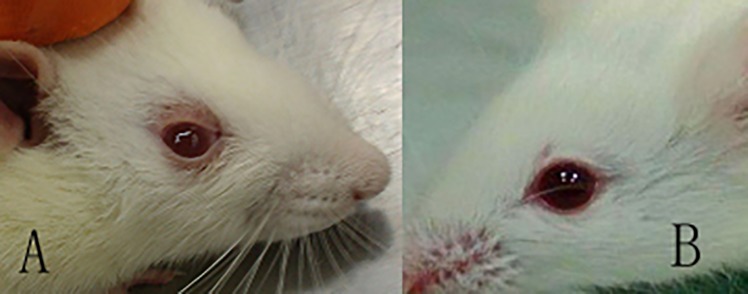
The periocular appearance of TAO and control rats. Red and swollen periocular tissues were observed in the TAO rats (A), while the control rats showed a normal appearance (B).

During the experimental period, we found that the periocular tissues of rats treated with I-131 and sodium levothyroxine (TAO rats) changed compared with those of the control rats. The tissues around the eyes of 14 TAO rats appeared red and swollen, as shown in Fig 1. However, no abnormal changes in the eyes were observed in the control rats.

### 3.2 Effect of exposure on weight and intraocular pressure of the rats

**Fig 2:**
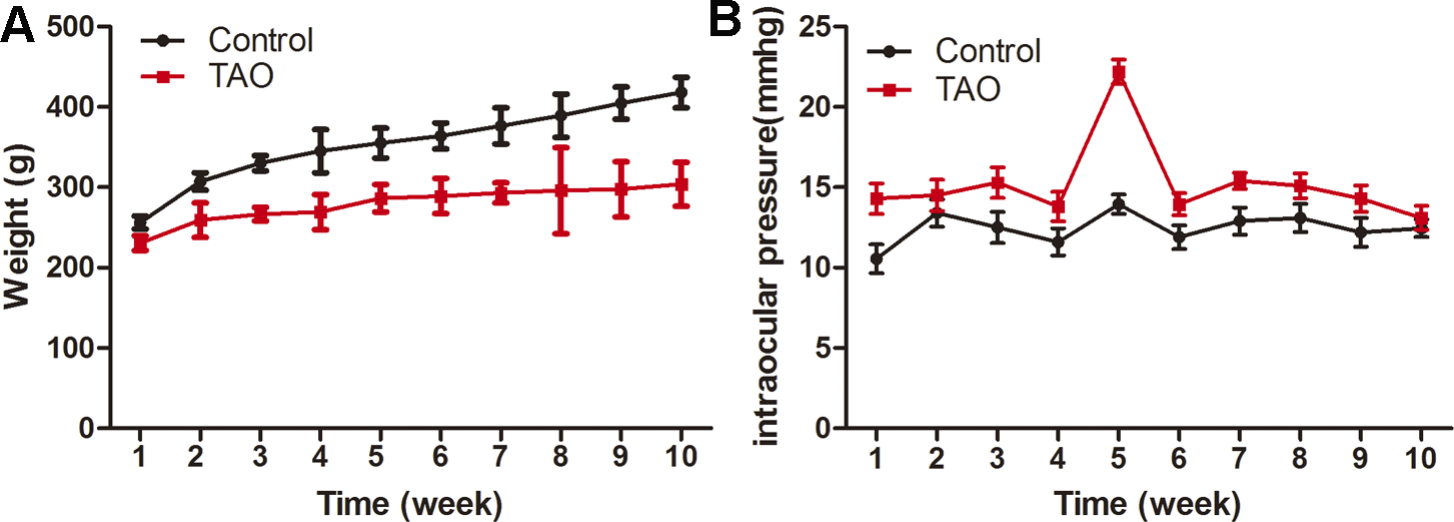
Changes in weight and intraocular pressure in TAO and control rats. The TAO rats showed a lower weight but a higher intraocular pressure compared with the control rats (n = 15).

The weight and intraocular pressure were measured over the entire period, and the results are displayed in Fig 2. The weight of the control rats showed an increasing trend, and the weight gain was faster than that of the TAO rats. The mean weight of the TAO rats was 279.14±22.56 g, which was significantly lower than that of the control rats (355.22±48.95 g). The intraocular pressure of the TAO rats fluctuated during the 10 weeks, peaking at week 5, with a mean value of 22.2±0.75 mmHg. However, the control group showed a relatively small change in intraocular pressure, with values ranging from 10.55±0.89 mmHg to 13.95±0.6 mmHg. Moreover, the TAO rats showed a statistical higher mean pressure (15.17±3.93 mmHg) than the control group (12.45±0.99 mmHg).

### 3.3 Effect of exposure on the serum levels of T3, T4, TSH and TRAb

**Fig 3:**
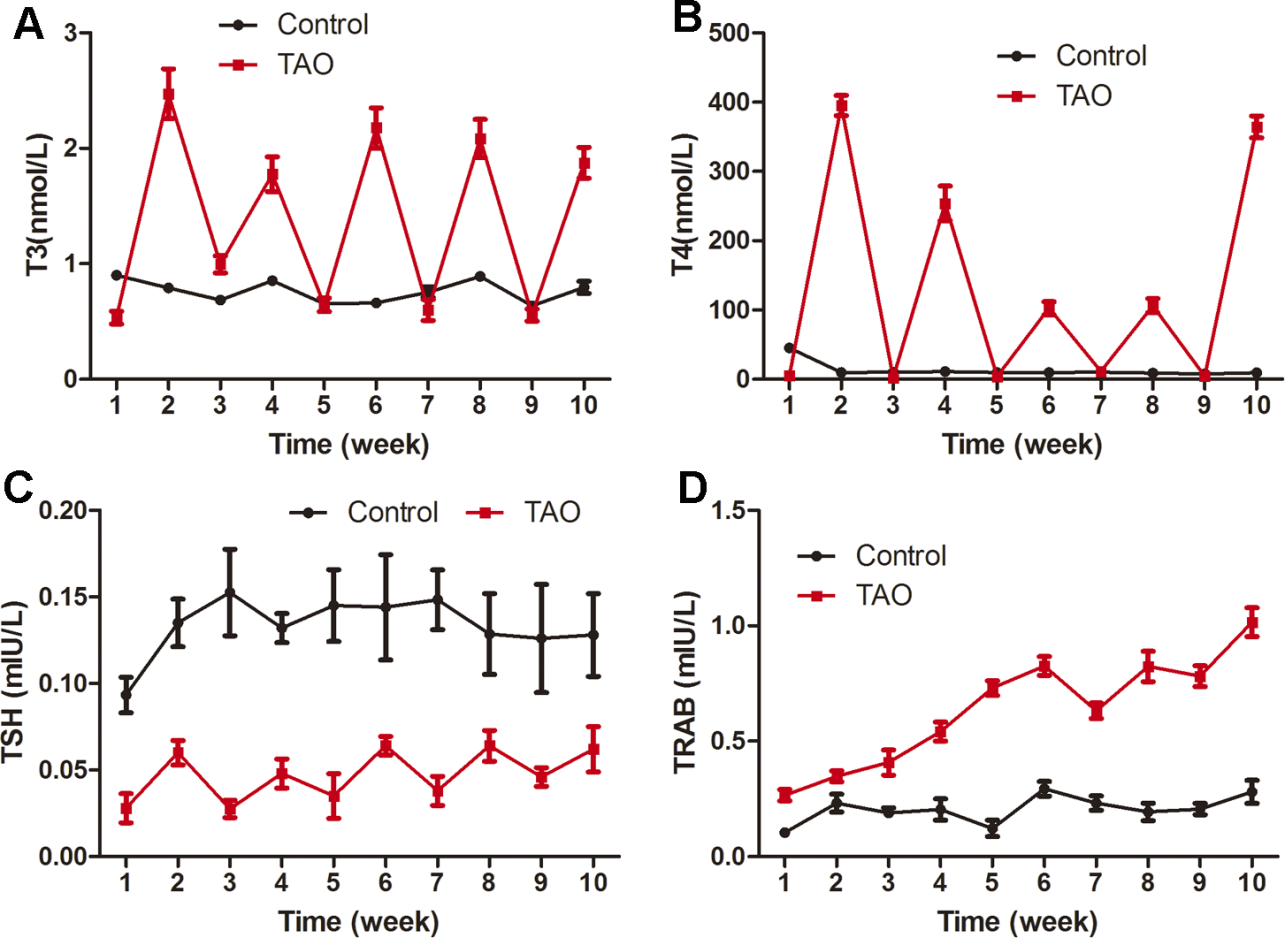
The serum T3, T4, TSH and TRAb levels of rats. The T3 and T4 indexes of the control rats showed a very small change during the 10 weeks, while those of the TAO rats displayed fluctuations according to the administration of I-131 and sodium levothyroxine. The TSH levels were lower in the treated rats compared with those in the controls, but the TRAb levels showed an up-regulating trend over the entire experimental period.

The serum levels of T3, T4, TSH and TRAb are given in Fig 3. The T3 and T4 levels exhibited fluctuations in the TAO group according to the administration of I-131 and sodium levothyroxine during the 10-week experimental period. The administration of I-131 can damage the thyroid and cause a lower secretion of T3 and T4, while the discontinuous administration of sodium levothyroxine can up-regulate the levels of those two indexes in drug-treated rats. However, in the control group, the T3 and T4 concentrations showed very small changes over the entire experimental period. Compared with the control group (0.76±0.1 nmol/L), the mean concentration of T3 was significantly up-regulated in the TAO group (1.37±0.77 nmol/L). The results for T4 were similar to those for T3, with a mean concentration of 12.79±1.64 nmol/L in the normal group and a much higher value of 124.24±154.9 nmol/L in the TAO group.

The TSH levels also displayed fluctuations during the 10-week study. The mean TSH level in the TAO group (0.047±0.01 mIU/L) was significantly lower than that in the control group (0.133±0.01 mIU/L). Regarding the TRAb level, the TAO group showed an increasing trend; at each time point, the values were significantly higher than those of the control group, while the control group exhibited only small changes over the entire experimental period.

### 3.4 Effect of exposure on pathological changes in tissues

**Fig 4:**
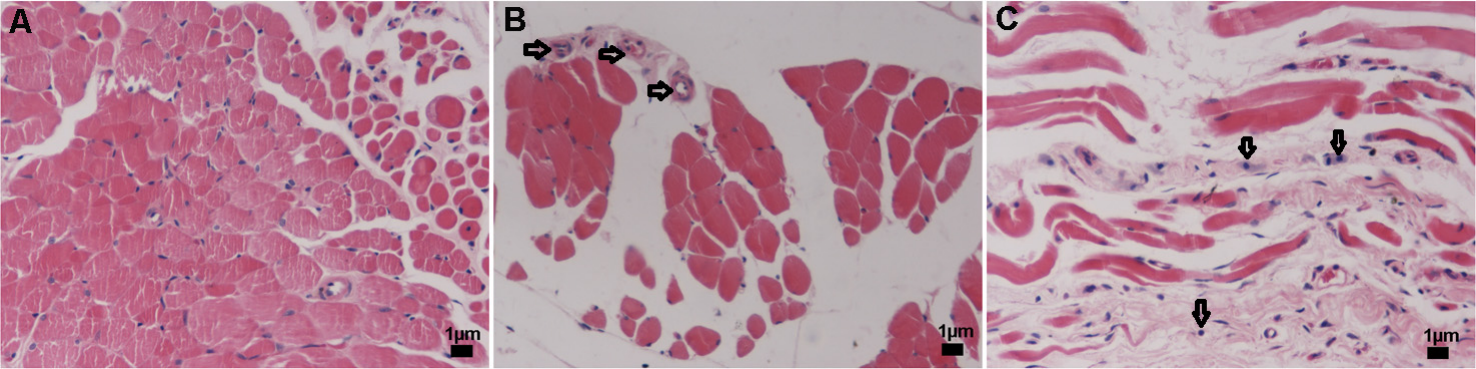
Pathological changes in the orbital tissues of the rats. No abnormality was found in the control rats (A), while increased numbers of blood vessels (B) and inflammatory cell infiltrations (C) were observed in the orbital tissues of the TAO rats. The right arrows indicate the blood vessels, and the downward arrows indicate inflammatory cells.

One of the characteristics of TAO is inflammatory cell infiltration in the orbital tissue. The H&E staining results for the orbital tissue sections are exhibited in Fig 4. There was no obvious infiltration of lymphocytes or other inflammatory cells in the control group (Fig 4A). However, in the TAO group, the number of inflammatory cells and blood vessels in the orbital tissue was higher compared with that in the control group (Fig 4B and 4C), a result that conforms to the TAO features.

### 3.5 Effect of exposure on tissue ultrastructure

**Fig 5:**
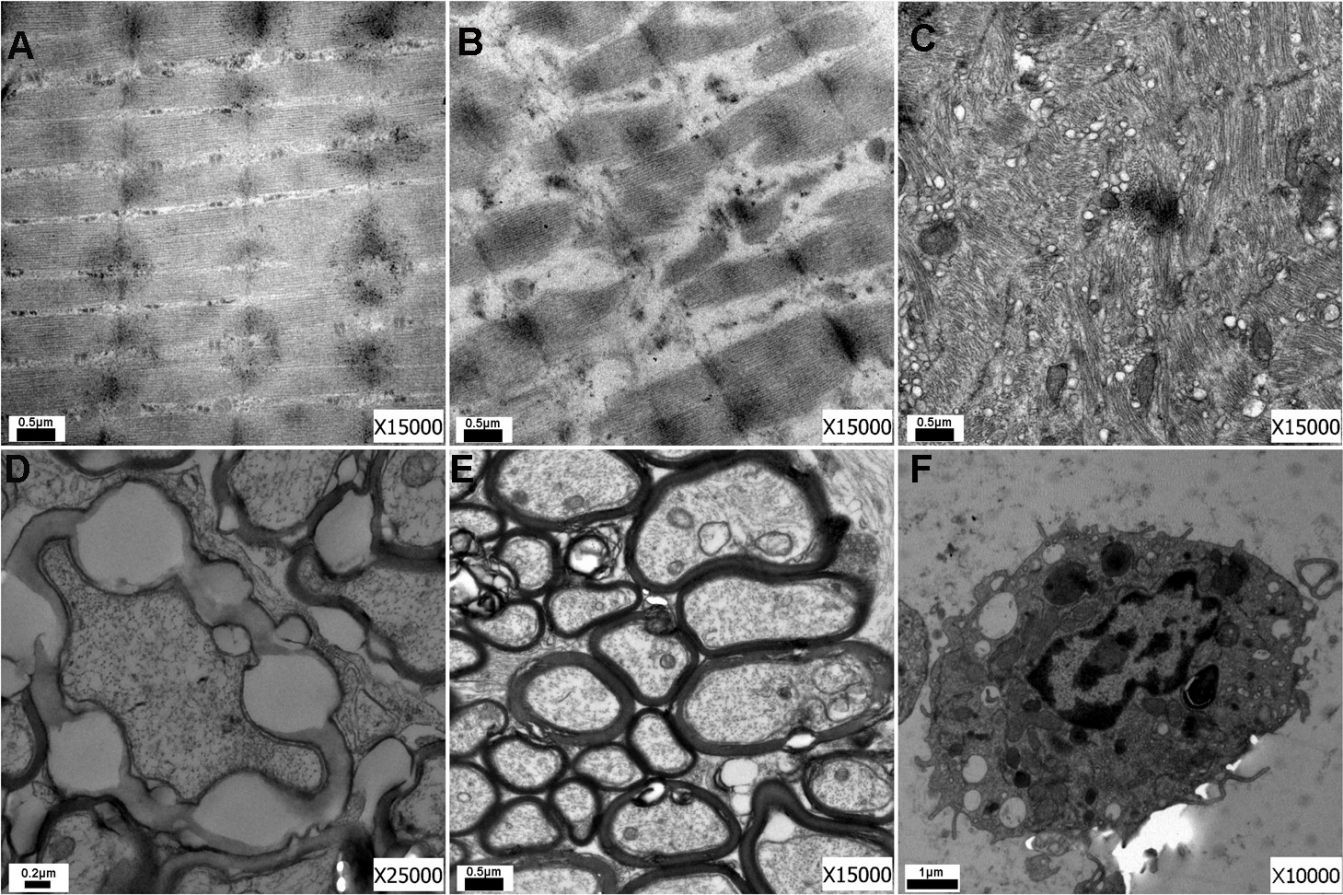
Ultrastructure of the orbital tissues in the rats. No abnormality was found in the control rats (A); however, the orbital muscle fiber in the TAO rats appeared fractured and dissolved (B). Twisted fibers, mitochondrial swelling and vacuoles within the endoplasmic reticulum (C) as well as macrophages (F) were found in the TAO group. Moreover, swelling nerve fibers (D) and shedding nerve myelin (E) were also observed in the TAO rats.

TEM images of ultrathin sections of orbital tissue from the rats are shown in Fig 5. The orbital tissue from the control rats showed a normal structure with a clearly visible Z-line (Fig 5A) in the muscle fibers, while a fractured and dissolved muscle was found (Fig 5B) with a twisted structure in the TAO rats (Fig 5C). Swollen and vacuolized mitochondria and a dilated endoplasmic reticulum, swelling nerve fibers and shedding nerve myelin were also observed in the TAO rats (Fig 5C, 5D and 5E). Moreover, the orbital tissue of the TAO rats was infiltrated with macrophages and other inflammatory cells (Fig 5F), confirming the HE staining results presented in Fig 4C.

## Discussion

In clinical practice, we observed that patients with hyperthyroidism after I-131 therapy often developed hypothyroidism and thyroid-associated ophthalmopathy with increased TRAb levels. Patients who have already developed TAO may show worsening of their disease with the administration of I-131. Studies have also shown that hyperthyroidism patients treated with I-131 can easily develop TAO [13, 16]. In this study, we treated SD rats with I-131 by gavage to damage their thyroids and cause hypothyroidism, followed by discontinuous supplementation of thyroid hormone (sodium levothyroxine). This regimen led to an abnormal fluctuation of thyroid hormone in the treated rats to simulate the clinical TAO process, with the aim of establishing a stable and easily obtained TAO animal model. Unlike other methods [7, 8, 10], we did not inject antibodies into experimental rats to mimic a diseased status of the animal; rather, the animals spontaneously produced antibodies and induced TAO.

I-131 is an isotope of iodine with a physical half-life of 7 days; gamma rays can be emitted for imaging, and beta rays can be emitted for treatment. Because of the need for iodine in thyroid tissue for the synthesis of thyroid hormone, I-131 can be absorbed by the thyroid. As the range of beta rays emitted by I-131 is only 1–2 mm, this radiation can only destroy cells that gather around the thyroid. The released energy will damage the thyroid follicular epithelial cells and further reduce the secretion of hormones, thereby achieving the treatment purpose. Because I-131 is primarily distributed in the thyroid, unabsorbed I-131 will be quickly excreted by the urinary system; therefore, no other damage will be produced [4, 17, 18]. In our study, the rats treated with I-131 exhibited hypothyroid status due to the destruction of their thyroids, as we can see in the results of T3 and T4, the TAO rats showed lower T3 and T4 levels when compared with the control rats at week 1. Meanwhile, these rats also showed lighter weight (Fig 1A) and some abnormal behaviors such as the motor retardation, sleepiness and poor appetite.

Early in 1995, Bartley and Gorman proposed diagnostic criteria for TAO [19]. If the patient exhibits eyeball retraction, a TAO diagnosis can be confirmed providing that one or all of the following signs are verified: (1) the serum levels of total T3 (TT3), FT4 (free thyroxine), TT4 (total T4), and free triiodothyronine (FT3) are elevated, and the levels of TSH are decreased; (2) the eyeball shows proptosis with a degree ≥20 mm, and the difference between the two eyes is ≥2 mm; (3) the extraocular muscles are affected with limited activity of the eyeball, and dilated extraocular muscles can be found by CT; (4) optic nerve dysfunction is observed, including decreased visual acuity, pupillary reflex, color vision and visual field abnormalities, and cannot be explained by other lesions. However, if the patient does not demonstrate eyeball retraction, for a diagnosis of TAO, they must show abnormal thyroid function with either exophthalmos, extraocular muscle involvement or optic neurological dysfunction, and other similar diseases should be ruled out. In the present study, we used SD rats to establish a TAO animal model. Although the orbital anatomy of rats is different from that of humans, with limitations on the observation of eyeball proptosis or eyelid retraction signs, other characteristics of TAO in rats still show great similarity to those in humans.

Weight, intraocular pressure and other results of gross observation are important signs for the diagnosis of TAO. Due to the infiltration of inflammatory cells and the dilation of blood vessels, the eyelid and conjunctiva of TAO patients often appear red and swollen [1–4], results that were confirmed in our study. TAO and autoimmune thyroid disease are closely related, and one of the typical features of autoimmune thyroid disease is metabolic syndrome with the symptoms of weight loss, fear of heat, sweating, and positive urine glucose findings [1–3]. Therefore, weight loss can be a diagnostic sign of TAO. Research has also shown that 76% of GO patients with exophthalmos display abnormal intraocular pressure, and a total of 24% of 500 TAO patients were found to have high intraocular pressure, ranging from 22 to 30 mmHg [20]. Sen et al. [21] compared the intraocular pressure of GO patients and healthy people and found that, although both intraocular pressure values were in the normal range, the TAO patients had a significantly higher value than the healthy patients. These studies suggest that an elevated intraocular pressure can also be a sign of TAO. In our study, we measured the weight and intraocular pressure at different time points and found that the weight was remarkably reduced while the intraocular pressure was obviously increased in the TAO group. These results are consistent with those of the above studies.

The regulation of T3, T4 and TSH in the human body relies on the hypothalamus- pituitary-thyroid axis. The pituitary gland is a biological sensor for thyroid hormone and regulates the TSH level according to the concentrations of FT4 and FT3. A lower level of thyroid hormones would stimulate the secretion of TSH, but elevated hormone levels can inhibit TSH secretion. The American Thyroid Association (ATA) recommends that the diagnosis of hyperthyroidism be established by elevated levels of FT4 combined with a decreased TSH concentration (<0.1 mIU/l) [22]. Therefore, it can be seen that the levels of thyroid hormone and TSH are equally important for the diagnosis of hyperthyroidism and TAO. In the Bartley diagnostic criteria, TAO patients have elevated serum levels of TT3, FT4, TT4, and FT3 and attenuated levels of TSH [23]. In the present study, the serum indexes of the TAO group showed trends similar to those of the above studies, further confirming the feasibility of our TAO animal model.

TRAb is a type of IgG immunoglobulin. Due to immune dysfunction in TAO patients, thyroid-stimulating hormone receptor antibodies (TRAb) will be produced and directly bond with the TSH receptor on the cell membrane. TRAb is a polyclonal antibody and can be divided into two types: thyroid-stimulating antibody (TSAB) and thyroid-blocking antibody (TSBAB) [24]. The serum TSAB detection rate of untreated Graves’ disease patients can be as high as 80–100% [25], and the serum TSAB titer in patients with concomitant eye disease is significantly higher than that of patients without eye disease [26]. In the absence of obvious clinical manifestations, the measurement of TRAb can be vital evidence for assessing hyperthyroidism [27, 28]. In the current study, with the extension of the experimental period, the TRAb levels of the rats in the TAO group were significantly increased compared to the control group, confirming the presence of an immune response in the experimental rats. Compared with the commonly used immune method of injecting TSHR proteins into the animals [29], we mimicked the clinical disease process in rats and successfully obtained an elevated TRAb level, a result that was considered to be caused by autoimmunity and should more closely match the natural course of the disease and be more similar to the human TAO.

Several studies have confirmed that edema and inflammatory infiltration can be found in orbital tissue, extraocular muscles and lacrimal glands from TAO patients, and pathological changes in the active and inactive stages of the disease are obviously different [1–4]. The inflammatory cells primarily consist of mast cells and lymphocytes. Moreover, collagen fibers and glycosaminoglycan are widely found in the extraocular muscle, which, to some extent, tear the tissues apart. Similarly, we also observed the existence of mast cells and lymphocytes in orbital tissue sections through H&E staining, further confirming that pathological changes occurred in the treated rats. Macrophages are also found in the orbital tissue, and the collagen fibers and organelles such as the mitochondria display abnormal changes in the orbital tissue of patients [1, 30]. In our study, we observed macrophages and phagocytic vacuoles in the ultrastructure of the orbital tissue of rats treated with I-131 with pathological changes in the organelles, such as dilatation of the endoplasmic reticulum and swelling and vacuolization of the mitochondria. All of the above evidence demonstrates the feasibility of establishing a TAO animal model by treatment with I-131 and sodium levothyroxine.

However, there remain limitations in this study, such as the changes in growth factors in orbital tissues from TAO patients [31–35] and the exclusion of diagnostic imaging. Additional studies are needed to further confirm changes in the treated animals.

## Conclusion

The Novel Thyroid Function Fluctuated Animal model induced by I-131 and sodium levothyroxine in this study exhibited characteristics similar to those of TAO patients in the clinic, providing an effective and simple method for establishing a stable animal model for research on the pathogenesis and prevention of TAO.
